# Health literacy and urban–rural disparities: a cross-sectional study of access, adherence, and quality of life across the neurological spectrum

**DOI:** 10.3389/fpubh.2026.1779801

**Published:** 2026-03-11

**Authors:** Yuan Yao, Tianyu Tang, Shidi Yang

**Affiliations:** 1 Department of Neurosurgery, The Affiliated Lihuili Hospital of Ningbo University, Ningbo, Zhejiang, China; 2 Department of Urology, The First Affiliated Hospital of Zhejiang University School of Medicine, Hangzhou, China

**Keywords:** China, health equity, health literacy, neurological diseases, quality of life, socioeconomic factors

## Abstract

**Background:**

Health literacy is critical for chronic disease self-management, yet remains understudied in neurological populations, particularly in low- and middle-income countries. This study examined health literacy prevalence, socioeconomic determinants, and their intersectional associations with healthcare utilization and quality of life among adults with chronic neurological conditions in China.

**Methods:**

A cross-sectional survey was conducted at a tertiary hospital from March 2022 to September 2025. Health literacy was assessed using the Brief Health Literacy Screen (BHLS). We used multivariable modified Poisson regression and intersectional analysis with multiplicative interaction terms to evaluate associations with healthcare access, emergency department utilization, medication adherence, and quality of life (EQ-5D-5L).

**Results:**

Among the 1,120 participants (mean age 57.0 years, 41.6% women), low health literacy prevalence was 33.6%, ranging from 28% in epilepsy to 68% in dementia. Education was the strongest predictor (adjusted prevalence ratio [aPR] 2.42), followed by rural residency (aPR 1.42), lowest income quintile (aPR 1.52), out-of-pocket financing (aPR 1.34), and digital exclusion (no smartphone: aPR 1.28). Low health literacy was independently associated with higher emergency department utilization (aPR 1.58), poor medication adherence (aPR 1.89), and lower quality of life (adjusted *β* − 3.7). These cross-sectional associations do not establish causal directionality, and reverse causation—whereby adverse health outcomes impair functional literacy—cannot be excluded. Cost barriers increased 2.5-fold from 6.9% in high to 17.3% in low literacy groups. Urban–rural residence and income demonstrated independent associations with outcomes, with no statistically significant multiplicative interactions detected, resulting in substantial cumulative disparities across socioeconomic strata.

**Conclusion:**

One-third of adults with neurological conditions in China have low health literacy, with pronounced socioeconomic and geographic disparities. These findings indicate that disadvantage accumulates through independent structural pathways, supporting multi-level interventions—including rural service expansion, universal health literacy precautions, and digital inclusion strategies—to achieve health equity.

## 1 Introduction

Chronic neurological conditions are among the leading causes of disability and death globally, with over 3.4 billion individuals affected worldwide, responsible for approximately 276 million disability-adjusted life years (DALYs) in 2019 (1, 2). Among these diseases, stroke is the second-leading cause of death globally, with 12.2 million new incident cases occurring annually, epilepsy affects around 50 million people worldwide, and dementia currently affects over 55 million people, a figure which is estimated to triple by 2050 (3, 4). The escalating prevalence of these conditions has profound implications for healthcare systems, particularly in low- and middle-income countries where 80% of the global neurological disease burden is concentrated, yet resources remain severely limited (5).

The management of chronic neurological conditions demands sustained patient engagement with complex healthcare systems, medication adherence, and rehabilitation services (6). This sustained engagement is particularly vulnerable to disruptions in overburdened health infrastructures, which disproportionately impact patients with chronic diseases (82–84). However, a patient’s ability to navigate these requirements is fundamentally shaped by health literacy—defined as “the cognitive and social skills which determine the motivation and ability of individuals to gain access to, understand, and use information in ways which promote and maintain good health” (7). Health literacy is conceptualized as a multidimensional construct encompassing functional literacy (basic reading and numeracy skills to understand health materials), communicative/interactive literacy (skills to extract information and derive meaning in different contexts), and critical literacy (ability to critically analyze information and use it to exert greater control over life events) (8, 9). The present study also focuses specifically on functional health literacy, representing the foundational dimension necessary for engagement with the healthcare system, though we acknowledge that communicative and critical dimensions may be equally or more important for sustained self-management in chronic neurological conditions. However, systematic reviews demonstrate that inadequate health literacy is associated with poorer health outcomes, including reduced medication adherence, higher hospitalization rates, and lower quality of life (10, 11). A meta-analysis of 96 studies found inadequate health literacy associated with 1.5-fold increased hospitalization and 1.75-fold increased mortality (12). In neurological populations, low health literacy has been linked to reduced medication adherence (56% vs. 78% in those with adequate literacy), increased emergency utilization, and poorer disease control (13, 14).

China bears a disproportionate share of the global neurological disease burden, a challenge exacerbated by rapid population aging. The country accounts for one-third of the world’s stroke burden, recording 3.9 million incident cases annually, alongside approximately 10 million individuals living with epilepsy and over 15 million with dementia (15–17). Traumatic brain injury prevalence has risen by 36% in 20 years, with 1.64 million hospital admissions in 2013 (18).

Despite this huge burden of disease, China’s healthcare system is beset with a number of serious structural challenges, which may be worsened by a lower level of health literacy. The fragmented health financing system, consisting of Urban Employee Basic Medical Insurance (UEBMI), Urban and Rural Resident Basic Medical Insurance (URRBMI), and out-of-pocket payments, generates large disparities in access and financial protection (19, 20). Geographical disparities are highly evident, and rural residents have significantly less access to specialist neurological care services and much greater delays in reaching the health facility and out-of-pocket healthcare costs compared to their urban counterparts (21, 22). A 2019 national survey found that only 29% of rural residents with chronic conditions had access to rehabilitation services, compared to 58% of urban residents. Critically, health literacy levels in China are still significantly lower than those of high-income countries, and only 23.1% of the Chinese population has adequate health literacy, which is far from the 36% target set by the Healthy China 2030 initiative (23, 24). Health literacy is especially poor among older adults, those with lower educational attainment, rural populations, and those with chronic diseases (25, 26). Although health literacy is increasingly recognized as a crucial social determinant of health, systematic investigations involving individuals with chronic neurological conditions remain scarce, particularly in the context of China’s rapidly aging population. While the literature on the international effects of inadequate health literacy on health outcomes in neurological populations is in place, critical knowledge gaps remain. First, previous studies have focused mainly on single neurological diseases with little comparative evidence on the patterns of health literacy among various neurological diseases, which are considered across the spectrum of chronic neurological diseases (26, 27, 85–87). Second, while there is abundant evidence on socioeconomic and geographical disparities in health outcomes in China, the mechanism by which health literacy, in conjunction with structural factors such as education, income, health financing, and digital access, affects healthcare utilization and quality of life is poorly understood (27, 28). Therefore, the current study addresses these knowledge gaps by examining health literacy and its relationships with healthcare access, utilization, and quality of life of adults with chronic neurological conditions in China. Specifically, we were interested in: (1) identifying sociodemographic, clinical, and health system factors linked to low health literacy, (2), examining the relationships between health literacy and healthcare utilization outcomes, (3) determining independent contributions of low health literacy to health related quality of life, and (4) examining the intersectional effects of urban–rural residence and income on health outcomes.

## 2 Methods

### 2.1 Study design and setting

This cross-sectional survey was conducted at the Department of Neurosurgery, The Affiliated Lihuili Hospital of Ningbo University in Ningbo, Zhejiang Province, China, from March 2022 to September 2025. The hospital provides comprehensive neurological services to urban, peri-urban, and rural populations, with specialized clinics for cerebrovascular disease, epilepsy, neurodegenerative disorders, neuro-oncology, neurotrauma, and neurodevelopmental conditions.

### 2.2 Participants and eligibility criteria

Eligible participants were adults (≥18 years) with physician-diagnosed chronic neurological conditions attending neurology clinics, rehabilitation services, or receiving inpatient care. Inclusion criteria required: (1) confirmed diagnosis of cerebrovascular disease (stroke/transient ischemic attack), epilepsy, neurodegenerative disease (dementia/Parkinson’s disease), traumatic brain injury, neuro-oncology (primary/metastatic brain tumors), neurodevelopmental disorder (cerebral palsy), or other chronic neurological condition; (2) diagnosis ≥6 months prior to enrollment; and (3) ability to provide informed consent or availability of a legally authorized representative for those with severe cognitive impairment. Exclusion criteria included acute neurological events within 4 weeks, terminal illness with life expectancy <6 months, severe psychiatric disorder precluding reliable responses, and inability to complete questionnaires despite proxy assistance.

### 2.3 Data collection and sampling strategy

We stratified sampling by neurological condition category (7 groups) and residence (urban/peri-urban/rural). Within each stratum, systematic random sampling was applied to daily clinic registration lists (e.g., selecting every third eligible patient) to minimize selection bias and ensure temporal representativeness. Sample size calculation assumed 30% low health literacy prevalence, 5% precision, 95% confidence level, and 10% non-response rate, requiring *n* = 1,018. We approached 1,287 eligible patients between March 2022 and September 2024; 98 declined participation (7.6% refusal rate; primary reasons: time constraints *n* = 52, privacy concerns *n* = 31, felt too unwell *n* = 15), 41 were excluded after enrollment (cognitive impairment precluding reliable responses despite proxy availability *n* = 23, incomplete questionnaire data *n* = 12, terminal illness discovered during interview *n* = 6), and 28 withdrew consent before completion. The final analytic sample comprised 1,120 participants, exceeding our target of 1,018 to ensure adequate power for stratified analyses and multivariable modeling. Participants who completed the study did not differ significantly from those who declined or withdrew regarding age (*p* = 0.41), sex (*p* = 0.68), or residence (*p* = 0.53), based on basic registration data available for non-participants. Trained research assistants administered structured questionnaires through face-to-face interviews in private settings. Interviews lasted 35–45 min. However, for the participants with apparent severe cognitive impairment (based on clinical diagnosis and inability to understand consent procedures), legally authorized representatives completed questionnaires on behalf of the patient. However, we did not administer standardized cognitive screening instruments (e.g., Mini-Mental State Examination) to objectively quantify cognitive status in all participants, which represents a limitation when interpreting health literacy findings, particularly in the dementia subgroup.

### 2.4 Measures and instruments

#### 2.4.1 Sociodemographic characteristics

Standardized items assessed age (years), sex at birth (male/female), residence (urban/peri-urban/rural), highest educational attainment (none/primary/secondary/college-university/postgraduate), employment status (employed/self-employed/unemployed/unable to work due to disability/retired/student), total monthly household income in Chinese Yuan (CNY) from all sources during the past 3 months. To account for household size, we calculated per capita household income by dividing total household income by the number of household members. Income quintiles (lowest/low/middle/high/highest) were derived from the distribution within our study sample rather than national benchmarks, as our sample represented patients accessing tertiary neurological care and may not reflect national income distributions. Quintile cutoffs were lowest (<2,500 CNY per capita/month), low (2,500–3,999), middle (4,000–5,999), high (6,000–8,499), and highest (≥8,500). Marital status, household size, and ethnicity were also documented.

#### 2.4.2 Health system access factors

Health financing type was categorized as Urban Employee Basic Medical Insurance (UEBMI), Urban and Rural Resident Basic Medical Insurance (URRBMI), private insurance, out-of-pocket payment, or mixed coverage. Travel time to the facility was recorded in minutes. Digital access was assessed via smartphone ownership (personal/shared/none) and internet access (regular/occasional/none).

#### 2.4.3 Clinical profile and disease characteristics

Neurological condition group was categorized as cerebrovascular (ischemic stroke/hemorrhagic stroke/transient ischemic attack), epilepsy (focal/generalized/unknown), neurodegenerative (Alzheimer’s disease/vascular dementia/Parkinson’s disease/other), traumatic brain injury (mild/moderate/severe), neuro-oncology (primary brain tumor/metastatic), neurodevelopmental (cerebral palsy), or other chronic neurological disorder. The year of first diagnosis was recorded to calculate disease duration. Acute neurological events in the past 12 months (yes/no) and current tobacco use (never/former/current) were documented. Physician-diagnosed comorbidities were assessed via the following checklist: hypertension, diabetes, heart disease, chronic kidney disease, Chronic Obstructive Pulmonary Disease/asthma, depression, and anxiety (yes/no for each). Comorbidity count was summed (range 0–7). Current use of long-term prescribed medications (yes/no), rehabilitation services received in the past 6 months (physiotherapy/occupational therapy/speech therapy/none), and assistive device use (walking aid/wheelchair/none/other) were recorded.

#### 2.4.4 Health literacy assessment

The Brief Health Literacy Screen (BHLS) assessed functional health literacy—specifically, basic reading comprehension and form completion abilities—via three validated items: (1) “How often do you need to have someone help you when you read instructions, pamphlets, or other written material from your doctor or pharmacy?” (2) “How often do you have problems learning about your medical condition because of difficulty understanding written information?,” and (3) “How confident are you in filling out medical forms by yourself?” The BHLS does not assess communicative/interactive literacy (skills to extract and use information through verbal exchanges with providers, particularly relevant for neurological patients navigating complex multi-disciplinary care) or critical literacy (capacity to critically appraise and act upon health information). These dimensions, described in Nutbeam’s tripartite framework, may be equally or more important than functional literacy for sustained self-management in chronic neurological conditions. Furthermore, BHLS scores demonstrated a strong inverse gradient across educational categories (54.6% low literacy in none/primary vs. 19.0% in postgraduate), raising the possibility of partial construct overlap between BHLS performance and educational attainment. Although education was retained as a separate covariate in multivariable models, residual collinearity may attenuate independent estimates for both predictors. Future studies should employ multidimensional instruments such as the Health Literacy Questionnaire (HLQ) or Newest Vital Sign (NVS) to capture the full literacy spectrum in neurological populations (29).

#### 2.4.5 Disability assessment

The World Health Organization Disability Assessment Schedule 2.0 (WHODAS 2.0) 12-item version measured activity limitations and participation restrictions (30). The study used the tool in accordance with the WHO user agreement. Participants rated difficulty performing activities across six domains over the past 30 days (1 = none to 5 = extreme). Scores were summed (range 12–60), with scores ≥42 indicating moderate-to-severe disability.

#### 2.4.6 Health-related quality of life

Health-related quality of life was assessed using the EuroQol 5-Dimension 5-Level (EQ-5D-5L) instrument, following registration with the EuroQol Research Foundation (31). The instrument comprises five dimensions (mobility, self-care, usual activities, pain/discomfort, and anxiety/depression) and the EQ Visual Analog Scale (EQ-VAS). The EQ-VAS records the respondent’s self-rated health on a vertical visual analog scale, where the endpoints are labelled ‘The best health you can imagine’ (100) and ‘The worst health you can imagine’ (0).

#### 2.4.7 Psychological distress screening

The Patient Health Questionnaire-2 (PHQ-2) screened for depression via two items assessing depressed mood and anhedonia over the past 2 weeks (0 = not at all to 3 = nearly every day; range 0–6). Scores ≥3 indicated a positive depression screen. The Generalized Anxiety Disorder-2 (GAD-2) screened for anxiety via two items assessing anxiety and uncontrollable worry (same response scale; range 0–6). Scores ≥3 indicated a positive anxiety screen (32).

#### 2.4.8 Healthcare utilization

Participants reported the number of visits to neurologist/specialist (past 3 months), primary care physician (past 3 months), and rehabilitation services (past 3 months). Emergency department visits (past 6 months) and hospital admissions (past 12 months) were recorded as counts. Missed scheduled appointments (past 3 months; yes/no) and usual source of care (one regular facility-doctor/multiple facilities/no regular source/emergency department as primary) were documented.

#### 2.4.9 Medication adherence

Medication adherence was assessed using a 4-item medication adherence scale (4-MAS) adapted from established medication-taking behavior measures. The instrument evaluates four domains of adherence behavior using binary yes/no responses: unintentional non-adherence (forgetting), intentional non-adherence related to carelessness, discontinuation when feeling better, and discontinuation when experiencing adverse effects. Items assess behaviors over the past 30 days. Poor adherence was defined as reporting ≥2 adherence problems, consistent with validated scoring approaches for brief adherence measures. This threshold has demonstrated adequate sensitivity and specificity in identifying non-adherent patients in chronic disease populations.

#### 2.4.10 Access barriers assessment

Access barriers were evaluated using the Five A’s framework: (1) Affordability: skipped care or medications due to cost in past 12 months (yes/no); (2) Availability: frequency of obtaining appointment within 2 weeks when needed (never/rarely/sometimes/often/always); (3) Accessibility: travel time to facility >60 min one-way (yes/no); (4) Accommodation: clinic hours incompatible with work/family obligations (yes/no); (5) Acceptability: felt disrespected or not listened to by providers (yes/no). Additional items assessed communication barriers affecting care quality (yes/no), perceived adequacy of disability-friendly services (5-point Likert scale: 1 = strongly agree to 5 = strongly disagree), and experienced discrimination in healthcare settings in the past 12 months (yes/no) (33).

#### 2.4.11 Financial burden and social support

Out-of-pocket healthcare spending in the past 30 days was recorded in Chinese Yuan (CNY). Participants reported whether they borrowed money or sold assets to pay for healthcare in the past 12 months (yes/no). Financial pressure from healthcare costs was assessed on a 5-point scale (1 = no pressure to 5 = extreme pressure). Moderate-extreme pressure was defined as a score ≥3. Perceived insurance coverage adequacy was rated as completely adequate, mostly adequate, somewhat adequate, or not at all adequate. Additionally, the living arrangement (lives alone; yes/no) and primary caregiver availability (spouse/adult child/other family/hired caregiver/none) were documented. For participants receiving caregiving assistance, the caregiver relationship and hours per week were recorded.

### 2.5 Statistical analysis

Continuous variables were summarized as mean ± standard deviation (SD) or median (interquartile range [IQR]) based on distribution normality assessed via the Shapiro–Wilk test and Q-Q plots. Categorical variables were presented as frequencies and percentages. Group comparisons used one-way ANOVA (normally distributed continuous), Kruskal-Wallis test (non-normally distributed continuous), or χ^2^ test (categorical). The Jonckheere-Terpstra test assessed linear trends across ordered categories (residence, education, income, health literacy).

Multivariable modified Poisson regression with robust variance estimated adjusted prevalence ratios (aPR) with 95% confidence intervals (CI) for binary outcomes, avoiding odds ratio overestimation when outcome prevalence exceeded 10% (34). Models adjusted for age, sex, residence, education, income, health financing, condition group, disability (WHODAS-12), comorbidity count, psychological distress (PHQ-2/GAD-2), and social support. Multivariable linear regression estimated adjusted *β* coefficients (95% CI) for continuous outcomes (EQ-VAS). Model diagnostics included variance inflation factor assessment (VIF < 5), residual normality checks, and goodness-of-fit testing.

Intersectional equity analyses examined urban–rural residence × income interactions using multiplicative interaction terms in regression models. Stratified analyses presented results by residence-income combinations when interaction *p*-values <0.10. Missing data patterns were examined. While missingness was <5% for most variables, educational attainment data were missing for 12.4% (*n* = 139) of participants. Consequently, while complete-case analysis was used for the primary approach, sensitivity analyses using multiple imputation with chained equations (m = 20 imputations) were prioritized to account for education-related missingness and minimize selection bias (35). All statistical tests were two-sided; *p* < 0.05 was considered statistically significant. Bonferroni correction applied for multiple comparisons within outcome families. Analyses used R version 4.3.1 (R Foundation for Statistical Computing, Vienna, Austria) with the following packages: tableone, gtsummary, sandwich, lmtest, DescTools, and mice.

## 3 Results

### 3.1 Sample characteristics

Of 1,120 participants (mean age 57.0 ± 16.3 years; 58.4% male), 57.4% resided in urban areas, 29.5% in rural areas, and 13.1% in peri-urban areas. The most common neurological conditions were cerebrovascular disease (45.7%), epilepsy (17.4%), neurodegenerative disorders (12.2%), and traumatic brain injury (10.4%). Educational attainment varied, with 17.5% having no formal or primary education, 30.7% secondary, and 39.4% possessing college or postgraduate degrees. Socioeconomically, 35.9% of households fell into the lowest or low-income quintiles. Health financing relied on URRBMI (34.6%) and UEBMI (27.9%), though 20.6% of participants paid primarily out-of-pocket. Travel time to healthcare facilities exceeded 60 min for 28.4% of the cohort.

Significant variations existed across condition groups (all *p* < 0.001). Participants with cerebral palsy were the youngest (29.5 ± 5.7 years), while those with dementia were the oldest (73.1 ± 7.8 years). Moderate-to-severe disability (WHODAS≥42) affected 49.6% of the total sample, with the highest rates in dementia (96.4%), cerebrovascular disease (66.8%), and traumatic brain injury (40.2%). Mental health burden was substantial: 52.5% screened positive for depression and 62.0% for anxiety. The prevalence of positive depression screens (indicating likely depressive disorder) was notably high among patients with dementia (75.9%), cerebrovascular disease (57.0%), and traumatic brain injury (47.0%). Overall, 35.9% of participants screened positive for both depression and anxiety (Table 1).

**Table 1:** Comprehensive sociodemographic and clinical characteristics by neurological condition group.

Characteristic	Overall	Cerebrovascular	Epilepsy	Dementia	Traumatic brain injury	Neuro-Oncology	Cerebral palsy	Other	*p* value
	(N = 1,120)	(n = 512)	(n = 195)	(n = 137)	(n = 117)	(n = 82)	(n = 54)	(n = 23)	
Sociodemographic characteristics									
Age, mean ± SD, y	57.0 ± 16.3	64.6 ± 9.8	41.7 ± 12.3	73.1 ± 7.8	48.1 ± 15.1	50.5 ± 12.6	29.5 ± 5.7	52.8 ± 13.4	<0.001
Male sex, No. (%)	654 (58.4)	322 (62.9)	107 (54.9)	60 (43.8)	72 (61.5)	45 (54.9)	30 (55.6)	18 (78.3)	0.001
Residence, No. (%)									
Urban	643 (57.4)	282 (55.1)	126 (64.6)	80 (58.4)	65 (55.6)	45 (54.9)	32 (59.3)	13 (56.5)	0.492
Peri-urban	147 (13.1)	76 (14.8)	19 (9.7)	17 (12.4)	19 (16.2)	6 (7.3)	7 (13.0)	3 (13.0)	
Rural	330 (29.5)	154 (30.1)	50 (25.6)	40 (29.2)	33 (28.2)	31 (37.8)	15 (27.8)	7 (30.4)	
Education level, No. (%)									
None/Primary	196 (20.0)	99 (22.7)	31 (16.7)	27 (24.1)	16 (15.8)	12 (16.0)	5 (9.6)	6 (33.3)	0.125
Secondary	344 (35.1)	146 (33.4)	59 (31.7)	43 (38.4)	38 (37.6)	34 (45.3)	20 (38.5)	4 (22.2)	
College/University	299 (30.5)	134 (30.7)	59 (31.7)	31 (27.7)	29 (28.7)	22 (29.3)	20 (38.5)	4 (22.2)	
Postgraduate	142 (14.5)	58 (13.3)	37 (19.9)	11 (9.8)	18 (17.8)	7 (9.3)	7 (13.5)	4 (22.2)	
Employment status, No. (%)									
Employed/Self-employed	392 (35.0)	105 (20.5)	128 (65.6)	3 (2.2)	61 (52.1)	48 (58.5)	34 (63.0)	13 (56.5)	<0.001
Unemployed	156 (13.9)	42 (8.2)	49 (25.1)	3 (2.2)	27 (23.1)	13 (15.9)	20 (37.0)	2 (8.7)	
Unable to work due to disability	15 (1.3)	11 (2.1)	1 (0.5)	0 (0.0)	2 (1.7)	0 (0.0)	0 (0.0)	1 (4.3)	
Retired	557 (49.7)	354 (69.1)	17 (8.7)	131 (95.6)	27 (23.1)	21 (25.6)	0 (0.0)	7 (30.4)	
Household income, No. (%)									
Lowest	170 (15.2)	96 (18.8)	12 (6.2)	26 (19.0)	17 (14.5)	8 (9.8)	5 (9.3)	6 (26.1)	<0.001
Low	232 (20.7)	117 (22.9)	32 (16.4)	28 (20.4)	25 (21.4)	19 (23.2)	5 (9.3)	6 (26.1)	
Middle	337 (30.1)	144 (28.1)	62 (31.8)	48 (35.0)	31 (26.5)	23 (28.0)	23 (42.6)	6 (26.1)	
High	214 (19.1)	92 (18.0)	49 (25.1)	19 (13.9)	23 (19.7)	20 (24.4)	9 (16.7)	2 (8.7)	
Highest	167 (14.9)	63 (12.3)	40 (20.5)	16 (11.7)	21 (17.9)	12 (14.6)	12 (22.2)	3 (13.0)	
Married, No. (%)	778 (69.5)	360 (70.3)	141 (72.3)	86 (62.8)	79 (67.5)	63 (76.8)	31 (57.4)	18 (78.3)	<0.001
Household size, mean ± SD	3.1 ± 1.2	3.1 ± 1.2	3.3 ± 1.1	2.9 ± 1.2	3.1 ± 1.2	3.1 ± 1.2	3.1 ± 1.2	3.4 ± 1.4	0.203
Health system access factors									
Health financing, No. (%)									
URRBMI	557 (49.7)	295 (57.6)	64 (32.8)	96 (70.1)	44 (37.6)	28 (34.1)	23 (42.6)	7 (30.4)	<0.001
UEBMI	321 (28.7)	113 (22.1)	83 (42.6)	6 (4.4)	49 (41.9)	43 (52.4)	17 (31.5)	10 (43.5)	
Out-of-pocket	94 (8.4)	48 (9.4)	11 (5.6)	16 (11.7)	11 (9.4)	1 (1.2)	3 (5.6)	4 (17.4)	
Private insurance	48 (4.3)	19 (3.7)	13 (6.7)	6 (4.4)	5 (4.3)	2 (2.4)	3 (5.6)	0 (0.0)	
Mixed	100 (8.9)	37 (7.2)	24 (12.3)	13 (9.5)	8 (6.8)	8 (9.8)	8 (14.8)	2 (8.7)	
Travel time to facility, median (IQR), min	32.5 (22.0–51.0)	33.0 (22.0–51.2)	32.0 (21.0–46.0)	35.0 (20.0–56.0)	31.0 (21.0–55.0)	34.5 (21.2–52.0)	31.0 (24.0–44.0)	27.0 (20.5–43.5)	<0.001
Personal smartphone access, No. (%)	801 (86.3)	356 (86.8)	136 (81.4)	95 (89.6)	82 (83.7)	69 (92.0)	45 (88.2)	18 (85.7)	0.002
Clinical profile and comorbidities									
Years since diagnosis, median (IQR)	3.0 (1.0–7.0)	2.0 (1.0–4.0)	7.0 (4.0–11.0)	2.0 (1.0–4.0)	5.0 (3.0–8.0)	3.0 (2.0–6.8)	26.5 (23.0–30.8)	4.0 (2.5–5.5)	<0.001
Acute event in past 12 months, No. (%)	280 (25.0)	166 (32.4)	31 (15.9)	44 (32.1)	21 (17.9)	13 (15.9)	4 (7.4)	1 (4.3)	<0.001
Current tobacco use, No. (%)	221 (19.7)	103 (20.1)	39 (20.0)	19 (13.9)	29 (24.8)	13 (15.9)	10 (18.5)	8 (34.8)	0.008
Comorbidities, No. (%)									
Hypertension	417 (37.2)	268 (52.3)	27 (13.8)	72 (52.6)	22 (18.8)	19 (23.2)	2 (3.7)	7 (30.4)	<0.001
Diabetes	205 (18.3)	133 (26.0)	11 (5.6)	40 (29.2)	10 (8.5)	7 (8.5)	0 (0.0)	4 (17.4)	<0.001
Heart disease	141 (12.6)	91 (17.8)	5 (2.6)	31 (22.6)	9 (7.7)	5 (6.1)	0 (0.0)	0 (0.0)	<0.001
Depression	154 (13.8)	70 (13.7)	20 (10.3)	37 (27.0)	15 (12.8)	7 (8.5)	5 (9.3)	0 (0.0)	<0.001
Anxiety	182 (16.2)	81 (15.8)	25 (12.8)	33 (24.1)	18 (15.4)	10 (12.2)	10 (18.5)	5 (21.7)	0.138
Comorbidity count, No. (%)									
0	405 (36.2)	119 (23.2)	120 (61.5)	18 (13.1)	60 (51.3)	42 (51.2)	36 (66.7)	10 (43.5)	<0.001
1	386 (34.5)	186 (36.3)	60 (30.8)	48 (35.0)	40 (34.2)	26 (31.7)	17 (31.5)	9 (39.1)	
2	215 (19.2)	136 (26.6)	13 (6.7)	37 (27.0)	11 (9.4)	13 (15.9)	1 (1.9)	4 (17.4)	
≥3	114 (10.2)	71 (13.9)	2 (1.0)	34 (24.8)	6 (5.1)	1 (1.2)	0 (0.0)	0 (0.0)	
Long-term prescribed medications, No. (%)	904 (80.7)	446 (87.1)	164 (84.1)	102 (74.5)	87 (74.4)	60 (73.2)	35 (64.8)	10 (43.5)	<0.001
Disability and mental health									
WHODAS-12 disability score (12–60), mean ± SD	40.7 ± 10.0	43.9 ± 7.1	28.9 ± 7.3	53.2 ± 4.7	39.1 ± 7.1	34.5 ± 7.9	35.9 ± 5.2	37.4 ± 5.6	<0.001
Moderate–severe disability (WHODAS ≥42), No. (%)	555 (49.6)	342 (66.8)	5 (2.6)	132 (96.4)	47 (40.2)	13 (15.9)	10 (18.5)	6 (26.1)	<0.001
Depression screen positive (PHQ-2 ≥ 3), No. (%)	588 (52.5)	292 (57.0)	70 (35.9)	104 (75.9)	55 (47.0)	33 (40.2)	23 (42.6)	11 (47.8)	<0.001
Anxiety screen positive (GAD-2 ≥ 3), No. (%)	694 (62.0)	334 (65.2)	98 (50.3)	113 (82.5)	66 (56.4)	39 (47.6)	29 (53.7)	15 (65.2)	<0.001
Both depression and anxiety screen positive, No. (%)	402 (35.9)	206 (40.2)	39 (20.0)	87 (63.5)	36 (30.8)	16 (19.5)	10 (18.5)	8 (34.8)	<0.001
Lives alone, No. (%)	96 (8.6)	46 (9.0)	9 (4.6)	23 (16.8)	13 (11.1)	4 (4.9)	0 (0.0)	1 (4.3)	0.018
Has primary caregiver, No. (%)	770 (100.0)	365 (100.0)	103 (100.0)	115 (100.0)	80 (100.0)	53 (100.0)	40 (100.0)	14 (100.0)	<0.001

CP, cerebral palsy; GAD-2, Generalized Anxiety Disorder 2-item scale; IQR, interquartile range; PHQ-2, Patient Health Questionnaire 2-item scale; TBI, traumatic brain injury; UEBMI, Urban Employee Basic Medical Insurance; URRBMI, Urban and Rural Resident Basic Medical Insurance; WHODAS, World Health Organization Disability Assessment Schedule. p values calculated using one-way ANOVA for normally distributed continuous variables, Kruskal-Wallis test for non-normally distributed continuous variables, and χ^2^ test for categorical variables. Education data is missing for 139 participants (12.4% of the sample). Caregiver availability was assessed only among participants reporting functional dependence or requiring assistance with activities of daily living; percentages, therefore, reflect the eligible subpopulation rather than the entire cohort.

### 3.2 Health literacy distribution and disparities

**Figure 1:**
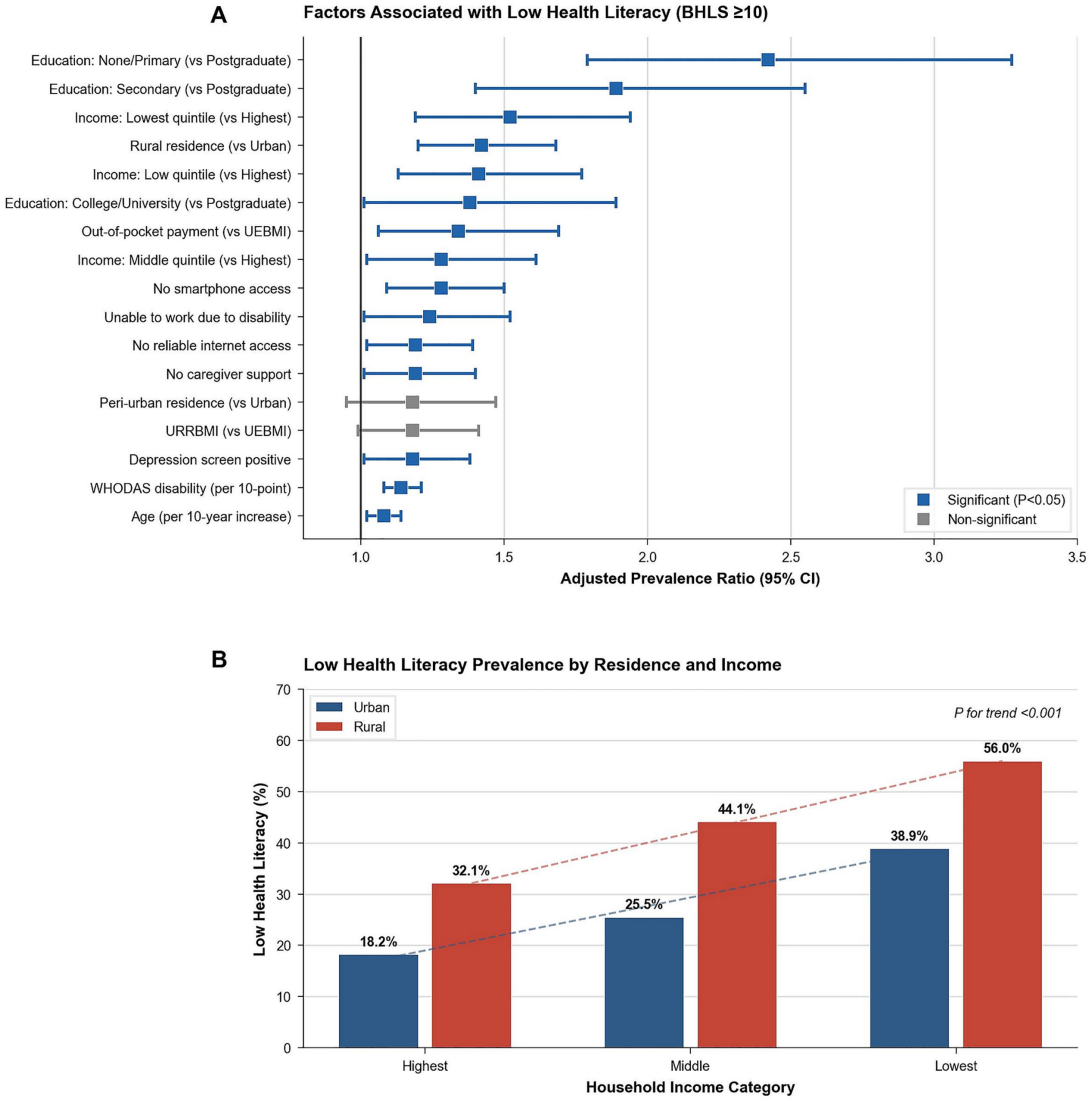
Social determinants of low health literacy among adults with chronic neurological conditions. **(A)** Adjusted prevalence ratios (aPRs) and 95% confidence intervals (CIs) for factors associated with low health literacy (BHLS ≥10), estimated using multivariable Poisson regression with robust variance. Models adjusted for sociodemographic, socioeconomic, clinical, and access-related covariates. **(B)** Prevalence of low health literacy stratified by residence (urban vs. rural) and household income category, demonstrating a clear socioeconomic gradient within both residential settings (*p* for trend <0.001).

Mean BHLS was 8.4 ± 2.4, with 33.6% (*n* = 376) categorized as low literacy (≥10), 44.3% moderate, and 22.1% high. Specific deficits included low confidence filling forms (52.7%) and difficulty understanding written materials (48.9%). Low literacy was significantly more prevalent in rural (44.2%) compared to peri-urban (31.3%) and urban (28.6%) areas (*p* < 0.001). Rural residents reported worse outcomes across all five health-related quality of life dimensions (all *p* < 0.001), with higher rates of mobility problems (68.8% vs. 48.2% urban) and anxiety/depression (79.1% vs. 58.7%). Consequently, poor QoL (EQ-VAS < 50) was higher in rural (54.5%) vs. urban (38.4%) settings (*p* < 0.001). Positive screens for depression (61.8% vs. 45.4%) and anxiety (71.5% vs. 56.0%) were also elevated among rural participants (*p* < 0.001) (Table 2; Figure 1B).

**Table 2:** Health literacy, disability, and quality of life by urban–rural residence.

Outcome	Urban	Peri-urban	Rural	*p* value	*p* trend
	(*n* = 643)	(*n* = 147)	(*n* = 330)		
Health literacy (BHLS)					
D1: How often needs help reading (1–5), mean ± SD	2.6 ± 1.3	2.9 ± 1.4	3.2 ± 1.3	<0.001	<0.001
D2: How often have problems understanding (1–5), mean ± SD	2.5 ± 1.4	2.8 ± 1.3	3.1 ± 1.4	<0.001	<0.001
D3: Confidence filling forms (1–5), mean ± SD	2.5 ± 1.4	2.8 ± 1.5	3.1 ± 1.4	<0.001	<0.001
BHLS composite score (3–15), mean ± SD	7.6 ± 2.4	8.5 ± 2.5	9.4 ± 2.5	<0.001	<0.001
Low health literacy (BHLS ≥10), No. (%)	184 (28.6)	46 (31.3)	146 (44.2)	<0.001	<0.001
Disability (WHODAS-12)					
WHODAS-12 score (12–60), mean ± SD	39.1 ± 10.3	41.2 ± 9.7	43.1 ± 9.2	0.002	0.001
Moderate–severe disability (≥42), No. (%)	283 (44.0)	80 (54.4)	192 (58.2)	0.003	0.001
Quality of life (EQ-5D-5L)					
Mobility problems (level 3–5), No. (%)	310 (48.2)	96 (65.3)	227 (68.8)	<0.001	<0.001
Self-care problems (level 3–5), No. (%)	390 (60.7)	99 (67.3)	232 (70.3)	<0.001	<0.001
Usual activities problems (level 3–5), No. (%)	468 (72.8)	118 (80.3)	271 (82.1)	<0.001	<0.001
Pain/discomfort (level 3–5), No. (%)	178 (27.7)	50 (34.0)	130 (39.4)	<0.001	<0.001
Anxiety/depression (level 3–5), No. (%)	377 (58.7)	96 (65.3)	261 (79.1)	<0.001	<0.001
EQ-VAS score (0–100), mean ± SD	58.3 ± 15.4	54.9 ± 16.1	50.1 ± 16.7	<0.001	<0.001
EQ-VAS < 50 (poor QoL), No. (%)	247 (38.4)	66 (44.9)	180 (54.5)	<0.001	<0.001
Psychological distress					
PHQ-2 score (0–6), mean ± SD	2.6 ± 2.0	2.8 ± 1.8	3.3 ± 1.9	<0.001	<0.001
PHQ-2 ≥ 3 (depression screen+), No. (%)	292 (45.4)	76 (51.7)	204 (61.8)	<0.001	<0.001
GAD-2 score (0–6), mean ± SD	3.1 ± 1.8	3.4 ± 1.8	3.6 ± 1.9	<0.001	<0.001
GAD-2 ≥ 3 (anxiety screen+), No. (%)	360 (56.0)	98 (66.7)	236 (71.5)	<0.001	<0.001
Both PHQ-2 and GAD-2 ≥ 3, No. (%)	196 (30.5)	53 (36.1)	153 (46.4)	<0.001	<0.001

Health literacy, disability, and quality of life by education level						
Outcome	None/Primary	Secondary	College/University	Postgraduate	*p* value	*p* trend
	(*n* = 196)	(*n* = 344)	(*n* = 299)	(*n* = 142)		
Low health literacy (BHLS ≥10), No. (%)	107 (54.6)	124 (36.0)	87 (29.1)	27 (19.0)	<0.001	<0.001
WHODAS-12 score, mean ± SD	47.2 ± 8.1	42.3 ± 9.2	38.7 ± 10.1	35.1 ± 10.9	<0.001	<0.001
EQ-VAS score, mean ± SD	45.2 ± 16.8	52.1 ± 15.9	58.3 ± 14.7	64.5 ± 13.2	<0.001	<0.001
Depression screen+ (PHQ-2 ≥ 3), No. (%)	134 (68.4)	201 (58.4)	156 (52.2)	50 (35.2)	<0.001	<0.001

Health literacy, disability, and quality of life by household income quintile						
Outcome	Lowest	Low	Middle	High	Highest	*p* value
	(*n* = 170)	(*n* = 232)	(*n* = 337)	(*n* = 214)	(*n* = 167)	
Low health literacy, No. (%)	82 (48.2)	89 (38.4)	107 (31.8)	59 (27.6)	39 (23.4)	<0.001
WHODAS-12 score, mean ± SD	46.8 ± 8.5	43.2 ± 9.1	40.1 ± 9.8	38.4 ± 10.3	34.7 ± 10.9	<0.001
EQ-VAS score, mean ± SD	46.3 ± 16.9	51.5 ± 16.2	55.8 ± 15.4	58.7 ± 14.8	62.8 ± 13.5	<0.001
Depression screen+, No. (%)	118 (69.4)	141 (60.8)	173 (51.3)	102 (47.7)	55 (32.9)	<0.001

BHLS, Brief Health Literacy Screen; EQ-5D-5L, EuroQol 5-Dimension 5-Level; EQ-VAS, EuroQol Visual Analog Scale; GAD-2, Generalized Anxiety Disorder 2-item scale; PHQ-2, Patient Health Questionnaire 2-item scale; QoL, quality of life; WHODAS, World Health Organization Disability Assessment Schedule. *p*-values calculated using one-way ANOVA for continuous outcomes and *χ*^2^ test for categorical outcomes. *p* for trend assessed using Jonckheere-Terpstra test across ordered categories.

Educational gradients were steep: low literacy decreased from 54.6% (none/primary) to 19.0% (postgraduate) (*p* < 0.001). Higher education correlated with lower disability scores (WHODAS-12: 47.2 to 35.1), reduced depression (68.4 to 35.2%), and higher EQ-VAS scores (45.2 to 64.5) (all *p* < 0.001) (Table 2). Income showed parallel associations: low literacy decreased from 48.2% (lowest quintile) to 23.4% (highest) (*p* < 0.001). From lowest to highest income quintiles, disability scores improved (46.8 vs. 34.7), EQ-VAS increased (46.3 vs. 62.8), and depression prevalence declined (69.4% vs. 32.9%) (all *p* < 0.001) (Table 2).

### 3.3 Predictors of low health literacy

Multivariable modified Poisson regression identified independent predictors: education (vs. postgraduate)—none/primary aPR 2.42 (95% CI 1.79–3.27, *p* < 0.001), secondary 1.89 (1.40–2.55, *p* < 0.001), college/university 1.38 (1.01–1.89, *p* = 0.045); rural residence (vs. urban) 1.42 (1.20–1.68, *p* < 0.001); income (vs. highest)—lowest quintile 1.52 (1.19–1.94, *p* = 0.001), low quintile 1.41 (1.13–1.77, *p* = 0.003), middle 1.28 (1.02–1.61, *p* = 0.035); out-of-pocket payment (vs. UEBMI) 1.34 (1.06–1.69, *p* = 0.014); no smartphone 1.28 (1.09–1.50, *p* = 0.002); no internet 1.19 (1.02–1.39, *p* = 0.027); WHODAS per 10-point 1.14 (1.08–1.21, *p* < 0.001); depression positive 1.18 (1.01–1.38, *p* = 0.037); no caregiver 1.19 (1.01–1.40, *p* = 0.034); unable to work 1.24 (1.01–1.52, *p* = 0.038); and age per 10-years 1.08 (1.02–1.14, *p* = 0.008). The model demonstrated strong explanatory power (Table 3; Figure 1A).

**Table 3:** Multivariable analysis: factors associated with low health literacy (BHLS ≥10).

Variable	Crude PR	*p* value	Adjusted PR	*p* value
	(95% CI)		(95% CI)	
Age (per 10-y increase)	1.15 (1.09–1.21)	<0.001	1.08 (1.02–1.14)	0.008
Female sex (vs. male)	1.23 (1.05–1.44)	0.012	1.14 (0.97–1.33)	0.105
Residence (ref: Urban)				
Peri-urban	1.32 (1.06–1.64)	0.014	1.18 (0.95–1.47)	0.138
Rural	1.68 (1.44–1.96)	<0.001	1.42 (1.20–1.68)	<0.001
Education (ref: Postgraduate)				
None/Primary	2.85 (2.12–3.83)	<0.001	2.42 (1.79–3.27)	<0.001
Secondary	2.14 (1.59–2.88)	<0.001	1.89 (1.40–2.55)	<0.001
College/University	1.52 (1.11–2.09)	0.009	1.38 (1.01–1.89)	0.045
Employment (ref: Employed/Self-employed)				
Unemployed	1.28 (1.04–1.58)	0.021	1.12 (0.91–1.38)	0.284
Unable to work due to disability	1.56 (1.29–1.89)	<0.001	1.24 (1.01–1.52)	0.038
Retired	1.31 (1.06–1.62)	0.013	1.09 (0.87–1.36)	0.457
Income (ref: Highest quintile)				
Lowest	1.89 (1.49–2.40)	<0.001	1.52 (1.19–1.94)	0.001
Low	1.64 (1.31–2.05)	<0.001	1.41 (1.13–1.77)	0.003
Middle	1.38 (1.10–1.73)	0.006	1.28 (1.02–1.61)	0.035
High	1.19 (0.92–1.54)	0.186	1.15 (0.89–1.49)	0.28
Health Financing (ref: UEBMI)				
URRBMI	1.42 (1.20–1.68)	<0.001	1.18 (0.99–1.41)	0.069
Out-of-pocket	1.68 (1.35–2.09)	<0.001	1.34 (1.06–1.69)	0.014
No smartphone access	1.52 (1.31–1.77)	<0.001	1.28 (1.09–1.50)	0.002
No reliable internet access	1.38 (1.19–1.60)	<0.001	1.19 (1.02–1.39)	0.027
WHODAS disability (per 10-point increase)	1.21 (1.15–1.28)	<0.001	1.14 (1.08–1.21)	<0.001
Comorbidity count ≥3 (vs. 0)	1.28 (1.04–1.58)	0.022	1.15 (0.93–1.42)	0.197
Depression screen+ (PHQ-2 ≥ 3)	1.34 (1.15–1.56)	<0.001	1.18 (1.01–1.38)	0.037
Lives alone	1.24 (1.05–1.47)	0.012	1.08 (0.91–1.28)	0.382
No caregiver support	1.31 (1.12–1.53)	0.001	1.19 (1.01–1.40)	0.034

CI, Confidence interval; PHQ-2, Patient Health Questionnaire 2-item scale; PR, prevalence ratio; UEBMI, Urban Employee Basic Medical Insurance; URRBMI, Urban and Rural Resident Basic Medical Insurance; WHODAS, World Health Organization Disability Assessment Schedule. Low health literacy is defined as a Brief Health Literacy Screen composite score ≥10. The adjusted model includes all variables shown, plus condition group and marital status. Prevalence ratios estimated using modified Poisson regression with robust variance. Model *R*^2^ = 0.42; *N* = 1,120.

### 3.4 Healthcare utilization by health literacy

Utilization increased across literacy categories (high/moderate/low): ED visits (6 months) 34.3% (*n* = 85/248), 27.4% (*n* = 136/496), 32.7% (*n* = 123/376) (*p* < 0.001, *p*-trend<0.001). Hospital admissions (12 months): 21.4% (*n* = 53/248), 27.0% (*n* = 134/496), 21.8% (*n* = 82/376) (*p* = 0.001, *p*-trend<0.001). Missed appointments (3 months): 19.0% (*n* = 47/248), 14.5% (*n* = 72/496), 13.6% (*n* = 51/376). Notably, we observed a statistical suppression effect regarding missed appointments. In unadjusted analyses, high health literacy participants paradoxically reported a higher frequency of missed appointments (19.0%) compared to the low literacy group (13.6%). However, after adjusting for potential confounders—particularly age and employment status—the direction of association reversed. In the multivariable model, low health literacy became a significant independent predictor of missed appointments (aPR 1.64, 95% CI 1.20–2.25), indicating that the raw data masked the true barrier presented by low literacy once competing risks were controlled.

**Figure 2:**
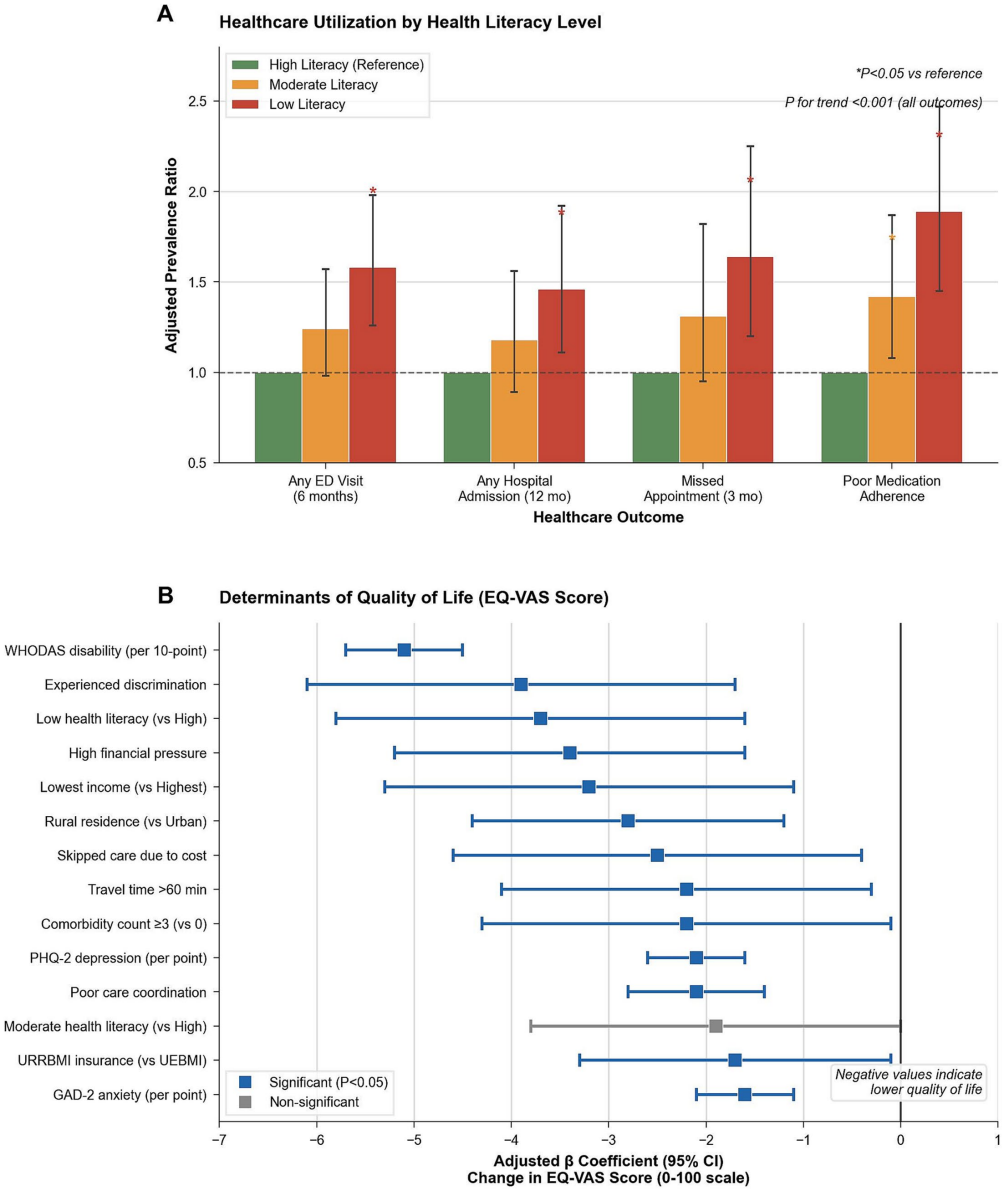
Association between health literacy and healthcare outcomes. **(A)** Adjusted prevalence ratios (aPRs) and 95% CIs for healthcare utilization outcomes—any emergency department visit (6 months), any hospital admission (12 months), missed appointments (3 months), and poor medication adherence—by health literacy level, with high literacy as the reference category. Models adjusted for sociodemographic, clinical severity, access barriers, and insurance status. **(B)** Multivariable linear regression coefficients (*β*) and 95% CIs for determinants of quality of life (EQ-VAS score, 0–100 scale). Negative coefficients indicate a lower quality of life. Significant predictors (*p* < 0.05) are highlighted.

Among 918 participants prescribed long-term medications, poor adherence (score ≥2) affected 17.3% (*n* = 35/202), 25.8% (*n* = 107/415), and 39.1% (*n* = 118/301) with high, moderate, and low literacy, respectively (*p* < 0.001, *p*-trend<0.001). Multivariable models adjusted for age, sex, residence, education, income, health financing, condition group, disability (WHODAS-12), comorbidity count, psychological distress (PHQ-2, GAD-2), and social support. Low vs. high literacy: ED visits aPR 1.58 (95% CI 1.26–1.98, *p* < 0.001), admissions 1.46 (1.11–1.92, *p* < 0.05), missed appointments 1.64 (1.20–2.25, *p* < 0.05), poor adherence 1.89 (1.45–2.47, *p* < 0.001). Moderate vs. high literacy: ED visits aPR 1.23 (0.98–1.54, *p* = 0.078), admissions 1.18 (0.89–1.57, *p* = 0.245), missed appointments 1.28 (0.91–1.81, *p* = 0.154), poor adherence 1.42 (1.08–1.87, *p* < 0.05). All models showed significant linear trends (all *p*-trend≤0.006). Model pseudo-R^2^ ranged 0.15–0.31 (Table 4; Figure 2A).

**Table 4:** Association between health literacy and healthcare utilization outcomes: multivariable analysis.

Health literacy level	Any ED visit aPR (95% CI)	Any hospital aPR (95% CI)	Missed aPR (95% CI)	Poor medication aPR (95% CI)
	(6 months)	Admission (12 months)	Appointment (3 months)	Adherence
High literacy (BHLS ≤6)	1.00 (ref)	1.00 (ref)	1.00 (ref)	1.00 (ref)
Moderate literacy (BHLS 7–9)	1.24 (0.98–1.57)	1.18 (0.89–1.56)	1.31 (0.95–1.82)	1.42 (1.08–1.87)^a^
Low literacy (BHLS ≥10)	1.58 (1.26–1.98)^a^	1.46 (1.11–1.92)^a^	1.64 (1.20–2.25)^a^	1.89 (1.45–2.47)^a^
*p* for trend across categories				
Linear trend *p*-value	<0.001	0.006	0.002	<0.001
Model *R*^2^				
Pseudo-*R*^2^ value	0.18	0.22	0.15	0.31

aPR, adjusted prevalence ratio; BHLS, Brief Health Literacy Screen; CI, confidence interval; ED, emergency department. aP < 0.05; all low literacy comparisons *p* < 0.001, moderate literacy medication adherence p < 0.05, other moderate literacy comparisons *p* > 0.05. All models adjusted for age, sex, residence (urban/peri-urban/rural), education, income quintile, health financing, condition group, WHODAS-12 disability score, comorbidity count, depression screen (PHQ-2 ≥ 3), anxiety screen (GAD-2 ≥ 3), and caregiver support. Prevalence ratios estimated using modified Poisson regression with robust variance. *N* = 1,120 for ED/admission/missed appointment models; N = 918 for adherence model (restricted to those prescribed long-term medications).

### 3.5 Access barriers and care quality

Five A’s barriers differed across literacy levels (high/moderate/low): affordability 6.9%/10.7%/17.3% (*p* = 0.007); availability 18.5%/24.6%/32.7% (*p* = 0.013); and acceptability 10.9%/15.5%/21.0% (*p* = 0.034). Accessibility (22.2%/27.0%/34.3%, *p* = 0.089) and accommodation (14.1%/18.5%/23.1%, *p* = 0.156) barriers also increased with lower literacy. Low literacy was associated with higher communication barriers (24.5% vs. 8.9% high, *p* < 0.001), inadequate disability services (52.4% vs. 31.5%, *p* = 0.002), and experienced discrimination (17.8%/10.3%/5.6%, *p* < 0.001).

### 3.6 Financial burden

Median monthly OOP spending increased with lower literacy: 325 CNY (IQR 198–725) high, 389 (215–850) moderate, 485 (268–1,050) low (*p* < 0.001). OOP > 500 CNY/month was reported by 28.6%/38.1%/47.3% across literacy groups (*p* < 0.001). Financial distress indicators also increased with lower literacy: borrowed money/sold assets 8.1%/13.5%/21.5% (*p* < 0.001); high financial pressure 24.2%/31.9%/42.3% (*p* < 0.001); and inadequate insurance coverage 35.5%/49.0%/61.2% (*p* < 0.001) (Table 5). Out-of-pocket spending increased inversely with literacy (325 to 485 CNY), highlighting financial vulnerability. Despite lower routine utilization, low literacy patients faced higher catastrophic costs (>500 CNY: 47.3% vs. 28.6%), suggesting that delaying care forces a shift from cost-effective prevention to expensive acute interventions.

**Table 5:** Comprehensive Healthcare Utilization, Access Barriers, and Financial Burden by Health Literacy Level.

Outcome	High literacy	Moderate literacy	Low literacy	*p* value	*p* trend
	(*n* = 248)	(*n* = 496)	(*n* = 376)		
Healthcare utilization (Past 12 Months)					
Neurology visits (past 3 months), median (IQR)	2.0 (1.0–3.0)	2.0 (1.0–3.0)	2.0 (1.0–3.0)	0.012	0.004
Any ED visit (past 6 months), No. (%)	85 (34.3)	136 (27.4)	123 (32.7)	<0.001	<0.001
Any hospital admission (past 12 months), No. (%)	53 (21.4)	134 (27.0)	82 (21.8)	0.001	<0.001
Missed appointment (past 3 months), No. (%)	47 (19.0)	72 (14.5)	51 (13.6)	0.042	0.015
Medication adherence (Past 30 Days)^a^					
Ever forget to take medicines? No. (%)	48 (23.8)	105 (25.3)	95 (31.6)	<0.001	<0.001
Careless about taking medicines, No. (%)	41 (20.3)	89 (21.4)	82 (27.2)	<0.001	<0.001
Stop medicines when feeling better, No. (%)	28 (13.9)	67 (16.1)	71 (23.6)	<0.001	<0.001
Stop medicines when feeling worse, No. (%)	32 (15.8)	73 (17.6)	78 (25.9)	<0.001	<0.001
Poor adherence (≥2 items “Yes”), No. (%)	35 (17.3)	107 (25.8)	118 (39.1)	<0.001	<0.001
Access barriers (Five A’s Framework)					
Affordability: Skipped care/meds due to cost, No. (%)	17 (6.9)	53 (10.7)	65 (17.3)	0.007	0.002
Availability: Cannot get an appointment within 2 weeks, No. (%)	46 (18.5)	122 (24.6)	123 (32.7)	0.013	0.004
Accessibility: Travel time > > 60 min, No. (%)	55 (22.2)	134 (27.0)	129 (34.3)	0.089	0.032
Communication barrier affected care, No. (%)	22 (8.9)	77 (15.5)	92 (24.5)	<0.001	<0.001
Experienced discrimination in healthcare, No. (%)	14 (5.6)	51 (10.3)	67 (17.8)	<0.001	<0.001
Financial burden and catastrophic expenditure					
Out-of-pocket spending (CNY/months), median (IQR)	325 (198–725)	389 (215–850)	485 (268–1,050)	<0.001	<0.001
OOP spending >500 CNY/month, No. (%)	71 (28.6)	189 (38.1)	178 (47.3)	<0.001	<0.001
Borrowed money or sold assets (12 months), No. (%)	20 (8.1)	67 (13.5)	81 (21.5)	<0.001	<0.001
Moderate-extreme financial pressure (score 3–5), No. (%)	60 (24.2)	158 (31.9)	159 (42.3)	<0.001	<0.001
Insurance coverage is inadequate, No. (%)	88 (35.5)	243 (49.0)	230 (61.2)	<0.001	<0.001

BHLS, Brief Health Literacy Screen; CNY, Chinese Yuan; ED, emergency department; IQR, interquartile range; OOP, out-of-pocket. Health literacy categories: High (BHLS ≤6), Moderate (BHLS 7–9), Low (BHLS ≥10). *p*-values calculated using the *χ*^2^ test for categorical outcomes and the Kruskal-Wallis test for continuous outcomes. ^a^ Medication adherence outcomes restricted to *n* = 918 participants prescribed long-term medications (High literacy *n* = 202, Moderate *n* = 415, Low *n* = 301).

### 3.7 Quality of life determinants

Mean EQ-VAS scores declined significantly with literacy level: 61.2 (high), 58.1 (moderate), and 48.5 (low) (*p* < 0.001). Poor QoL (EQ-VAS < 50) prevalence doubled from 25.4% in the high literacy group to 50.5% in the low literacy group (*p* < 0.001). In multivariable regression, disability (WHODAS-12) was the strongest predictor of lower QoL (adjusted *β* − 5.1 per 10-points, *p* < 0.001). Low health literacy (*β* − 3.7 vs. high, *p* = 0.001) and psychological distress (PHQ-2 *β* − 2.1; GAD-2 *β* − 1.6) were significant independent determinants. Other negative predictors included experienced discrimination (*β* − 3.9), high financial pressure (*β* − 3.4), lowest income quintile (*β* − 3.2), rural residence (*β* − 2.8), skipping care due to cost (*β* − 2.5), and travel time >60 min (*β* − 2.2). Clinical factors such as comorbidities (*β* − 2.2) and recent ED visits (*β* − 1.8) also reduced scores. The full model explained 58% of the variance in quality of life (Table 6; Figure 2B).

**Table 6:** Multivariable linear regression analysis: determinants of quality of life (EQ-VAS Score).

Variable	Crude β	*p* value	Adjusted β	*p* value
	(95% CI)		(95% CI)	
Health literacy (ref: High [BHLS ≤6])				
Moderate literacy (BHLS 7–9)	−3.8 (−6.2 to −1.4)	0.002	−1.9 (−3.8 to 0.0)	0.051
Low literacy (BHLS ≥10)	−8.6 (−11.2 to −6.0)	<0.001	−3.7 (−5.8 to −1.6)	0.001
Sociodemographic factors				
Age (per 10-y increase)	−1.2 (−1.8 to −0.6)	<0.001	−0.4 (−0.9 to 0.1)	0.132
Female sex (vs. male)	−2.1 (−4.0 to −0.2)	0.031	−1.3 (−2.8 to 0.2)	0.084
Rural residence (vs. urban)	−5.9 (−7.9 to −3.9)	<0.001	−2.8 (−4.4 to −1.2)	0.001
None/Primary education (vs. Postgraduate)	−6.2 (−9.1 to −3.3)	<0.001	−2.4 (−4.9 to 0.1)	0.058
Lowest income (vs. highest)	−7.4 (−9.9 to −4.9)	<0.001	−3.2 (−5.3 to −1.1)	0.003
Unemployed (vs. employed)	−4.8 (−7.1 to −2.5)	<0.001	−1.8 (−3.7 to 0.1)	0.067
Not married (vs. married)	−3.4 (−5.5 to −1.3)	0.002	−1.4 (−3.1 to 0.3)	0.109
Health system factors				
URRBMI insurance (vs. UEBMI)	−4.1 (−6.1 to −2.1)	<0.001	−1.7 (−3.3 to −0.1)	0.041
Travel time >60 min to facility	−5.6 (−8.0 to −3.2)	<0.001	−2.2 (−4.1 to −0.3)	0.024
Clinical and disability factors				
WHODAS disability (per 10-point increase)	−6.8 (−7.4 to −6.2)	<0.001	−5.1 (−5.7 to −4.5)	<0.001
PHQ-2 score (per point, 0–6)	−3.9 (−4.4 to −3.4)	<0.001	−2.1 (−2.6 to −1.6)	<0.001
GAD-2 score (per point, 0–6)	−3.2 (−3.7 to −2.7)	<0.001	−1.6 (−2.1 to −1.1)	<0.001
Comorbidity count ≥3 (vs. 0)	−5.3 (−7.9 to −2.7)	<0.001	−2.2 (−4.3 to −0.1)	0.043
Any ED visit (past 6 months)	−4.7 (−6.7 to −2.7)	<0.001	−1.8 (−3.4 to −0.2)	0.031
Access barriers and financial factors				
Skipped care due to cost (12 months)	−6.3 (−8.9 to −3.7)	<0.001	−2.5 (−4.6 to −0.4)	0.018
Experienced discrimination in healthcare	−7.8 (−10.5 to −5.1)	<0.001	−3.9 (−6.1 to −1.7)	0.001
High financial pressure (score 3–5)	−8.1 (−10.2 to −6.0)	<0.001	−3.4 (−5.2 to −1.6)	<0.001
Social Support				
No caregiver support	−4.2 (−6.3 to −2.1)	<0.001	−1.7 (−3.4 to 0.0)	0.052
Lives alone	−3.6 (−5.9 to −1.3)	0.002	−1.2 (−3.1 to 0.7)	0.213

BHLS, Brief Health Literacy Screen; CI, confidence interval; ED, emergency department; EQ-VAS, EuroQol Visual Analog Scale; GAD-2, Generalized Anxiety Disorder 2-item scale; PHQ-2, Patient Health Questionnaire 2-item scale; UEBMI, Urban Employee Basic Medical Insurance; URRBMI, Urban and Rural Resident Basic Medical Insurance; WHODAS, World Health Organization Disability Assessment Schedule. *β* coefficients represent the mean difference in EQ-VAS score (0–100 scale, higher scores indicate better quality of life). The adjusted model includes all variables shown plus the condition group. Model *R*^2^ = 0.58; adjusted *R*^2^ = 0.56; F-statistic = 42.3 (*p* < 0.001); *N* = 1,120.

### 3.8 Intersectional analysis: residence × income

**Figure 3:**
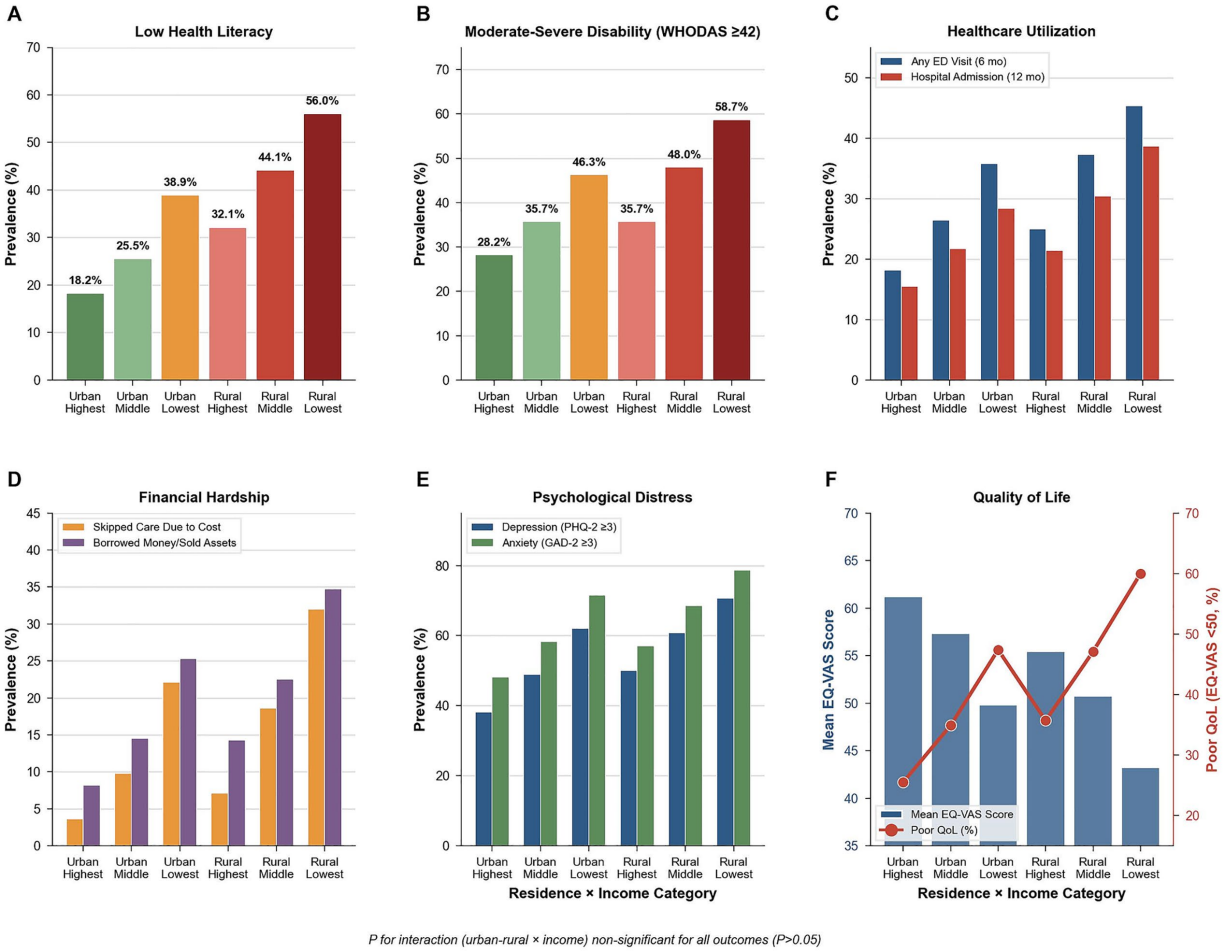
Intersectional equity analysis: Health outcomes by urban–rural residence and income. P</irevalence of adverse health and healthcare outcomes across combined categories of residence (urban vs. rural) and household income (highest, middle, lowest): **(A)** Low health literacy, **(B)** Moderate–severe disability (WHODAS ≥42), **(C)** Healthcare utilization (any emergency department visit in 6 months; any hospital admission in 12 months), **(D)** Financial hardship indicators (skipped care due to cost; borrowing money or selling assets), **(E)** Psychological distress (PHQ-2 ≥ 3; GAD-2 ≥ 3), and **(F)** Quality of life (mean EQ-VAS score and prevalence of poor quality of life, EQ-VAS < 50). Across all outcomes, gradients indicate cumulative disadvantage with decreasing income and rural residence. Tests for multiplicative interaction between residence and income were non-significant for all outcomes (*p* > 0.05).

Intersectional analysis revealed independent main effects of residence and income, with no evidence of multiplicative interaction across examined outcomes. Low literacy increased from 18.2% (urban-highest income) to 25.5%/38.9% (urban-middle/lowest), 24.1%/32.4%/44.0% (peri-urban-highest/middle/lowest), to 32.1%/44.1%/56.0% (rural-highest/middle/lowest) (*p*-interaction = 0.892) (Figures 1B, 3A). Moderate–severe disability ranged 28.2–46.3% urban, 35.7–58.7% rural (*p*-interaction = 0.743). ED visits: 18.2–45.3% (*p*-interaction = 0.956); admissions: 15.5–38.7% (*p*-interaction = 0.832) (Figures 3B,C). Financial barriers: skipped care 3.6–32.0% (*p*-interaction = 0.715); borrowed money 8.2–34.7% (*p*-interaction = 0.802); OOP median 278–625 CNY; high pressure 18.2–51.3% (Figure 3D). Psychological distress: depression 38.2–70.7% (*p*-interaction = 0.789); anxiety 48.2–78.7% (*p*-interaction = 0.823) (Figure 3E). Quality of life: EQ-VAS 61.2–43.2 (*p*-interaction = 0.621); poor QoL 25.5–60.0%, with consistent 20–25 percentage point income gradients across residence strata (Figure 3F). All 12 outcomes showed non-significant interactions (*p* > 0.05), demonstrating residence and income operate through independent additive pathways. This pattern suggests interventions targeting either geographic or socioeconomic barriers may benefit diverse subgroups (Table 7; Figures 3A–F).

**Table 7:** Intersectional equity analysis: key outcomes stratified by urban–rural residence and income level.

Outcome	Urban residents (*n* = 643)			Rural residents (*n* = 330)			*p* for interaction
	Highest	Middle	Lowest	Highest	Middle	Lowest	
	Income	Income	Income	Income	Income	Income	
	(*n* = 110)	(*n* = 235)	(*n* = 95)	(*n* = 28)	(*n* = 102)	(*n* = 75)	
Low health literacy (BHLS ≥10), %	18.2	25.5	38.9	32.1	44.1	56	0.892
WHODAS-12 score ≥42 (moderate–severe disability), %	28.2	35.7	46.3	35.7	48	58.7	0.743
Any ED visit (6 months), %	18.2	26.4	35.8	25	37.3	45.3	0.956
Any hospital admission (12 months), %	15.5	21.7	28.4	21.4	30.4	38.7	0.832
Adherence based on 4-item scale, %	24.5	32.8	44.2	35.7	46.1	57.3	0.687
Skipped care due to cost (12 months), %	3.6	9.8	22.1	7.1	18.6	32	0.715
Experienced discrimination in healthcare (12 months), %	6.4	9.8	17.9	10.7	15.7	22.7	0.891
Borrowed money or sold assets (12 months), %	8.2	14.5	25.3	14.3	22.5	34.7	0.802
EQ-VAS score (0–100), mean	61.2	57.3	49.8	55.4	50.7	43.2	0.621
EQ-VAS < 50 (poor QoL), %	25.5	34.9	47.4	35.7	47.1	60	0.758
Depression screen positive (PHQ-2 ≥ 3), %	38.2	48.9	62.1	50	60.8	70.7	0.789
Anxiety screen positive (GAD-2 ≥ 3), %	48.2	58.3	71.6	57.1	68.6	78.7	0.823

BHLS, Brief Health Literacy Screen; ED, emergency department; EQ-VAS, EuroQol Visual Analog Scale; GAD-2, Generalized Anxiety Disorder 2-item scale; PHQ-2, Patient Health Questionnaire 2-item scale; QoL, quality of life; WHODAS, World Health Organization Disability Assessment Schedule. Income categories: Highest (highest and high quintiles combined), Middle (middle quintile), Lowest (low and lowest quintiles combined). *p* for interaction tests whether income gradient differs between urban and rural settings using multiplicative interaction terms in Poisson/linear regression models adjusted for age, sex, education, condition group, and disability.

## 4 Discussion

This cross-sectional study of 1,120 adults with chronic neurological conditions in China revealed that one-third (33.6%) had low health literacy, with pronounced socioeconomic and geographic disparities. Multivariable analysis identified education as the strongest predictor of low health literacy (adjusted prevalence ratio 2.42 for none/primary vs. postgraduate education), followed by rural residence (aPR 1.42), lowest income quintile (aPR 1.52), out-of-pocket healthcare financing (aPR 1.34), and lack of digital access (aPR 1.28 for no smartphone). Low health literacy was independently associated with a 58% higher prevalence of emergency department visits, 89% higher poor medication adherence, and 3.7-point lower quality of life scores after comprehensive adjustment. Given the cross-sectional design, these should be interpreted as associations rather than causal estimates; longitudinal research is required to confirm directionality. Intersectional analysis demonstrated that urban–rural residence and income operated through additive rather than multiplicative pathways, with low health literacy prevalence ranging from 18.2% among urban-highest income participants to 56.0% among rural-lowest income participants, but no significant interactions emerged (all *p* > 0.05). In addition, current findings pertain specifically to functional health literacy as measured by the BHLS, representing foundational abilities to read medical materials and complete forms. This constitutes an important measurement constraint. In neurological populations navigating complex, evolving treatment regimens, communicative literacy—the ability to engage productively in verbal exchanges with providers, extract and apply information in changing circumstances, and coordinate across multiple specialists—may be a more clinically proximal determinant of outcomes such as medication adherence and care coordination than functional literacy alone. Critical literacy, enabling patients to evaluate treatment options, question provider recommendations, and advocate for themselves within fragmented health systems, becomes increasingly important across longer disease trajectories. The BHLS is also positively correlated with educational attainment (Pearson *r* typically 0.45–0.60 in chronic disease populations), which creates partial collinearity in models including both predictors and may lead to underestimation of both education and health literacy effects. Research employing multidimensional instruments—such as the HLQ’s nine-domain structure—would enable examination of which literacy dimensions most strongly predict specific outcomes (treatment adherence vs. care navigation vs. shared decision-making) and would provide more nuanced guidance for targeted interventions. However, Nutbeam’s conceptual framework distinguishes functional, communicative/interactive, and critical dimensions of health literacy, which may exhibit distinct patterns and differential importance in neurological populations. Functional literacy provides foundational access to written health information, but communicative literacy—the ability to extract information through verbal exchanges and apply it in changing circumstances—may be particularly critical for neurological patients navigating complex rehabilitation protocols and evolving symptom management. Critical literacy, the capacity to critically analyze health information and use it to exert control over health decisions, becomes essential for long-term self-advocacy in fragmented healthcare systems. In neurological populations, the relative importance of these dimensions may shift across disease trajectories: functional literacy may be paramount during initial diagnosis and treatment initiation, while communicative and critical literacy become increasingly vital for sustained self-management and care coordination. Future research employing multidimensional health literacy instruments could elucidate which literacy dimensions most strongly predict specific outcomes (e.g., treatment adherence vs. care navigation vs. shared decision-making) across neurological condition types. Such evidence would enable more targeted interventions, for instance, simplified written materials for functional literacy deficits, versus communication skills training for interactive literacy challenges, versus decision aid development for critical literacy enhancement.

Our finding of 33.6% low health literacy prevalence is substantially higher than the 23.1% inadequate health literacy reported in China’s general adult population, but lower than the 47–54% inadequate literacy documented in international stroke cohorts (13, 36). This intermediate prevalence likely reflects our mixed neurological sample, which included conditions with varying cognitive impacts. Our prevalence aligns closely with a 2022 systematic review reporting 31–42% low health literacy among chronic disease populations in China (37, 38), suggesting neurological conditions confer similar literacy challenges to other chronic diseases rather than uniquely elevated deficits.

The condition-specific variation we observed—with the highest apparent low literacy prevalence in dementia (68%) and lowest in epilepsy (28%)—parallels patterns from stroke literacy studies. However, an important interpretive caveat applies to the dementia finding: without concurrent standardized cognitive screening, the 68% prevalence cannot be attributed solely to literacy deficits. Cognitive impairment intrinsic to dementia pathology—including impaired reading comprehension, reduced working memory, and executive dysfunction—directly compromises performance on the BHLS items regardless of pre-morbid literacy. The true prevalence of functional health literacy deficits in dementia patients, distinct from cognitive impairment, likely requires longitudinal assessment with premorbid literacy proxies (e.g., years of education, occupational literacy demands) or assessment during early disease stages before significant cognitive decline. Results in the dementia subgroup should therefore be interpreted with particular caution (38). However, our epilepsy findings may reflect measurement differences: we assessed functional health literacy (ability to read medical materials and complete forms) rather than disease-specific knowledge. Functional literacy may be better preserved in epilepsy patients who typically have intact cognitive function compared to stroke or dementia populations (39).

The steep educational gradient we identified—low literacy decreasing from 54.6% in those with none/primary education to 19.0% in postgraduate-educated participants—demonstrates stronger associations than reported in most international studies. This represents a 2.9-fold difference across educational categories, highlighting education as the most powerful predictor of health literacy in our model (40, 41). The European Health Literacy Survey found that education was a strong determinant of health literacy across populations (42), and systematic reviews indicate that education explains significant variance in health literacy (12). Similarly, our comprehensive multivariable model, in which education was the strongest individual predictor, explained 42% of the total variance in health literacy. This amplified effect in China likely reflects the country’s rapid educational expansion: individuals aged 60 + (who comprise the majority of neurological patients) experienced dramatically different educational opportunities than younger cohorts, creating sharper literacy gradients than in countries with more gradual educational development (43).

Our finding that rural residence independently predicted a 42% higher risk of low health literacy (aPR 1.42) after adjusting for education and income extends previous Chinese research documenting urban–rural health disparities. National surveys found rural residents had substantially higher odds of inadequate health literacy (25, 44). The persistence of rural disparities after comprehensive adjustments suggests additional mechanisms beyond measured confounders, potentially including differential healthcare quality, provider communication patterns, or community health literacy environments (45). Similarly, the income gradient showed low literacy declining from 48.2% in the lowest income quintile to 23.4% in the highest quintile, representing a 2.1-fold difference. The parallel gradients observed across education and income—each demonstrating approximately 2–3-fold differences—support a model of cumulative disadvantage arising from independent structural factors rather than synergistic interaction.

The independent effect of digital access—with lack of smartphone (aPR 1.28) and internet access (aPR 1.19) predicting low health literacy—represents a novel finding in neurological populations. The mechanism likely operates bidirectionally: low baseline literacy limits the ability to use digital health resources, while a lack of digital access restricts opportunities to build health knowledge (46). This finding has particular urgency given China’s rapid digitalization of healthcare delivery, including smartphone-based appointment systems in many hospitals (47).

Our finding that low health literacy independently predicted 58% higher emergency department utilization (aPR 1.58) and 89% higher poor medication adherence (aPR 1.89) after comprehensive adjustment aligns with international evidence but demonstrates larger effect sizes. A landmark systematic review by Berkman et al. found that inadequate health literacy was associated with increased hospitalization and emergency department use (10), and Miller’s meta-analysis reported that health literacy significantly predicted medication adherence (11). Our stronger associations may reflect China’s fragmented primary care system, where individuals with low literacy face greater difficulty navigating referral pathways and rely disproportionately on emergency departments for episodic care (19, 48).

Our adherence findings demonstrate a clear literacy gradient across all four measured behaviors. Individual items showed 17.3% overall poor adherence in high literacy participants, increasing to 39.1% in low literacy participants, representing a 2.3-fold difference. This discrepancy aligns with but slightly exceeds international estimates and likely reflects China’s healthcare context, where patients must coordinate multiple medication sources, payment systems, and follow-up appointments across fragmented delivery systems (49, 50). Notably, we found significant literacy gradients across all five A’s access domains, with cost barriers increasing 2.5-fold from 6.9% in high literacy to 17.3% in low literacy groups. Paradoxically, while low literacy participants reported higher cost barriers (17.3%), their median out-of-pocket spending was also higher (485 CNY vs. 325 CNY), suggesting they face both greater need and greater financial burden. This pattern likely reflects delayed care-seeking leading to more expensive acute interventions rather than cost-effective preventive care. This comprehensive demonstration of the Five A’s framework in neurological populations extends previous work. Chinese health services surveys found cost barriers affecting substantial proportions of rural residents with chronic disease (51, 52), but did not assess literacy as a predictor.

The multivariable quality of life model (*R*^2^ = 0.58) revealed disability as the dominant predictor (*β* − 5.1 per 10-point), with low health literacy showing a modest independent effect (*β* − 3.7, approximately one-third the magnitude of disability), challenging assumptions that literacy primarily drives quality of life in neurological populations. This pattern aligns with stroke literature, where disability explains the largest proportion of QoL variance (53–55), suggesting disability overwhelms other factors in determining patient-reported outcomes.

However, the indirect effects of health literacy through mediating pathways (access barriers, adherence, care coordination) likely amplify its total impact beyond the direct association. Our finding that discrimination experienced in healthcare settings independently predicted 3.9-point lower EQ-VAS scores highlights an underrecognized mechanism. Research on discrimination and health outcomes has documented significant associations with both physical and mental health (56, 57). The mechanism likely operates through both psychological stress pathways and behavioral responses (avoiding care, reduced adherence, mistrust of providers) (28).

Our intersectional analysis revealed that urban–rural residence and income operated through additive rather than multiplicative mechanisms across all 12 examined outcomes, with no significant interactions (all *p* > 0.05). This finding contrasts with studies documenting synergistic effects of race and socioeconomic position on health literacy in the United States (58, 59). The observed additive pattern—where disadvantage accumulates linearly without statistically significant multiplicative interaction—likely reflects the distinct structural nature of inequality in China. While the statistical interaction was non-significant, the cumulative burden is clinically profound: as shown in the intersectional analysis, the prevalence of low health literacy escalates from 18.2% in the most advantaged group (urban, highest income) to 56.0% in the most disadvantaged (rural, lowest income). This 3-fold absolute difference suggests that while rural residence and poverty may operate through independent pathways, they stack to create a ‘double jeopardy’ for vulnerable patients. Unlike intersectional frameworks in racialized societies where specific subgroups face synergistic marginalization, our findings suggest a ‘stacking’ of disadvantages in the Chinese context. Consequently, a rural patient with low income faces the mathematical sum of both penalties, resulting in the profound disparities observed (56.0% low literacy vs. 18.2%) (60, 61).

The additive pattern has important implications for intervention design. Synergistic interactions would necessitate targeted programs addressing multiple disadvantages simultaneously. Additive effects suggest single-dimension interventions (e.g., telemedicine to reduce rural access barriers, or subsidies to reduce cost barriers) may yield benefits across diverse population subgroups (62, 63). This aligns with China’s recent policy emphasis on universal health system strengthening rather than targeted programs for specific disadvantaged groups (64, 65).

However, the absolute magnitude of cumulative disadvantage remains striking: rural-lowest income participants showed 3.1-fold higher low literacy prevalence (56.0% vs. 18.2%), 2.1-fold higher disability (58.7% vs. 28.2%), and 2.4-fold higher poor quality of life (60.0% vs. 25.5%) compared to urban-highest income participants. These cumulative gaps exceed those documented in most high-income countries. The European Health Literacy Survey found lower differences in health literacy across combined socioeconomic categories, suggesting China’s health equity challenges, while additive in mechanism, remain more severe in magnitude than most developed nations (42, 66).

Several mechanisms likely explain the persistent rural–urban disparities we observed. First, healthcare infrastructure remains concentrated in urban centers: China has substantial urban–rural disparities in neurologist availability and specialized neurological services. Wu et al. documented that China bears one-third of the global stroke burden, with significant geographic disparities in specialist access (15, 67). Second, the quality differential extends beyond access—rural providers receive less continuing education and have fewer diagnostic resources, potentially affecting the quality of patient education and communication (68). Third, rural-to-urban migration for healthcare generates cascading challenges: patients face unfamiliar urban hospital systems, language barriers, temporary housing costs, and separation from social support networks (69, 70).

Our finding that travel time >60 min independently predicted 2.2-point lower quality of life scores (*β* − 2.2, *p* = 0.024) provides quantitative evidence for this access burden. The smaller effect in our sample may reflect selection bias—patients enrolling at our tertiary center have already overcome travel barriers, representing a higher-functioning subset of rural neurological patients. Three aspects of our findings warrant discussion of potential alternative explanations. First, female sex was non-significant in multivariable models (aPR 1.14, *p* = 0.105) after adjustment, contrasting with international evidence of higher low literacy risk among women (71, 72). This null finding may reflect China’s gender-specific educational history: while older Chinese women received less formal schooling, they often managed household health decisions and accumulated practical health knowledge, potentially offsetting educational disadvantages. Alternatively, survival bias may operate—women with very low literacy and severe neurological conditions may have higher mortality and thus be underrepresented in our cross-sectional sample (73–75). Second, our finding that URRBMI insurance (covering rural and urban residents without formal employment) independently predicted only a 1.7-point lower quality of life compared to UEBMI (employee insurance) contrasts with prior studies reporting larger differences. This may reflect recent policy improvements: China implemented URRBMI benefit expansions in 2018–2020, narrowing the gap with UEBMI (70, 76). Alternatively, unmeasured confounding may persist—URRBMI enrollees differ systematically from UEBMI members in employment stability, social capital, and health behaviors beyond income and education. Third, the absence of interaction effects contradicts theoretical frameworks positing synergistic disadvantage (77). However, methodological factors may explain this. Power to detect interactions is substantially lower than for main effects—our sample may be underpowered to detect modest interactions despite adequate power for main effects. Additionally, interaction patterns may differ for biological outcomes versus patient-reported outcomes we examined.

Findings suggest multi-level interventions. At policy levels, expanding rural neurological services, subsidizing specialist telemedicine, and strengthening primary care’s role in chronic disease management could reduce geographic disparities. Digital health literacy programs targeting older adults could leverage China’s smartphone penetration to improve health information access (47, 78). Simplifying healthcare financing and reducing fragmentation across UEBMI/URRBMI systems would reduce navigation complexity, particularly for low-literacy patients (79, 80). Clinically, findings support universal health literacy precautions in neurology care: using teach-back methods, providing illustrated medication schedules, assessing patient comprehension routinely, and connecting patients with care coordinators (80, 81). The pronounced care coordination problems we documented (affecting 35.4% of low literacy patients) suggest that investing in patient navigators could yield substantial benefits. The additive rather than multiplicative interaction pattern suggests that single-dimension interventions (e.g., mobile health clinics reducing rural access barriers, or pharmaceutical subsidies reducing cost barriers) may benefit diverse patient subgroups rather than requiring complex multi-targeted programs. However, the cumulative magnitude of disadvantage among rural-lowest income participants (56% low literacy, 59% severe disability, 60% poor quality of life) indicates that while single interventions may help, comprehensive multi-sectoral approaches remain necessary to achieve health equity.

This study offers several methodological strengths. The large, diverse sample (*n* = 1,120) spanning seven neurological condition categories provides comprehensive representation across the neurological disease spectrum. We employed validated instruments with established psychometric properties in Chinese populations (BHLS, EQ-5D-5L, WHODAS-2.0, PHQ-2/GAD-2) and systematic stratified sampling by condition and residence to ensure representativeness. Rigorous multivariable modeling using modified Poisson regression avoided odds ratio overestimation for common outcomes, with extensive covariate adjustment addressing major confounders. The intersectional analysis with multiplicative interaction terms represents methodological advancement over simple stratification, while multiple imputation (m = 20) addressed education-related missingness (12.4%). Comprehensive outcome assessment spanning utilization, adherence, access barriers, and quality of life provides an integrated understanding of health literacy impacts.

However, important limitations warrant acknowledgment. A primary limitation is the absence of validated cognitive screening (e.g., MMSE, MoCA), particularly affecting the dementia subgroup. Because the BHLS tool requires cognitive skills progressively impaired by dementia, the observed 68% low health literacy rate likely conflates genuine premorbid literacy deficits with disease-related cognitive decline. This conflation may artificially inflate apparent literacy deficits and bias outcome associations. Although partially mitigated by proxy respondents for severe cases, adjustments for WHODAS-12 disability, and persistent associations across neurological groups, these measures cannot replace direct cognitive testing. Future studies must pair literacy tools with cognitive assessments to isolate their independent effects. Importantly, sensitivity analyses excluding the dementia subgroup (n = 137, 12.2%) yielded broadly consistent results, indicating that our primary findings are not solely driven by this cognitive confounding. Second, and critically, the cross-sectional design precludes causal inference. Reverse causation is a substantial methodological concern: poorer quality of life, greater functional disability, and psychological distress may impair the cognitive resources and motivation required for health literacy, creating a bidirectional feedback loop that cross-sectional analysis cannot resolve. Specifically, patients with severe disability (49.6% had WHODAS ≥42) or active depression (52.5% positive screen) may have demonstrated reduced BHLS performance not because of pre-existing literacy deficits but because of current illness burden. Similarly, social isolation resulting from neurological disease may restrict access to health information environments that maintain literacy. Future prospective cohort studies with repeated literacy assessments at pre-defined clinical milestones—diagnosis, post-acute rehabilitation, chronic stabilization—are needed to establish temporal precedence and causal direction. Residual confounding by unmeasured cognitive factors, health beliefs, and prior healthcare experiences also cannot be excluded despite extensive covariate adjustment. Third, single tertiary-hospital recruitment introduces selection bias that limits external validity and likely produces conservative estimates. Accessing our facility requires overcoming substantial sequential barriers (e.g., referrals, travel, costs, digital navigation), which systematically exclude marginalized patients with lower literacy, greater poverty, and remote rural residence. Consequently, our data likely underestimates the true community burden and attenuates disparities. Specifically, our observed 33.6% overall low literacy prevalence, the rural–urban literacy disparity (aPR 1.42), BHLS-outcome associations (e.g., aPR 1.58 for ED visits), and the striking 56.0% low literacy rate in the rural-lowest income group likely all underrepresent the true population-level magnitude. Future research requires population-based sampling, primary care partnerships, or community outreach to accurately capture the health literacy burden across the full neurological disease spectrum. Specific implications: (a) the 33.6% prevalence may represent only the upper distribution—community-based studies might reveal >50% prevalence in rural neurological populations; (b) associations between low literacy and adverse outcomes may be conservative estimates, as the most severely affected individuals are systematically excluded; (c) the modest 42% rural residence effect (aPR 1.42) may underestimate true urban–rural disparities, as rural patients in our sample had already overcome geographic barriers; (d) financial burden estimates may be underestimated, excluding those unable to afford tertiary care. Future research employing community-based sampling, mobile clinic outreach to remote areas, or partnerships with primary care facilities would provide more representative estimates. Fourth, self-report measures of utilization, adherence, and access barriers lack validation against electronic medical records or administrative databases. Social desirability bias may lead to underreporting of non-adherence in face-to-face interviews, potentially attenuating true associations. Recall bias is particularly concerning for utilization data spanning 6–12 months, as cognitively impaired patients (49.6% had moderate–severe disability) may inaccurately remember visit frequencies. Partial mitigation factors include: (a) structured recall aids and specific timeframes to enhance accuracy, (b) emergency visits/hospitalizations are salient events less prone to recall failure, and (c) trained interviewers in private settings may have reduced social desirability bias compared to provider-administered surveys. Nevertheless, validation against objective data would likely reveal stronger associations than documented. Fifth, the BHLS assesses only functional literacy (reading/form completion), not communicative or critical dimensions potentially more important for sustained self-management. Residual confounding persists despite extensive adjustment—unmeasured factors (cognitive function, health beliefs, provider communication quality) may influence associations. Findings from Zhejiang Province may not generalize to other Chinese regions with different healthcare infrastructure or socioeconomic profiles.

## 5 Conclusion

Low health literacy represents a pervasive correlate of health inequities among adults with chronic neurological conditions in China, deeply entrenched within gradients of education, rurality, and poverty. The independent and cumulative nature of these disparities—operating through additive rather than multiplicative pathways—suggests that single-dimension interventions can benefit diverse subgroups, though comprehensive approaches remain necessary. Achieving health equity demands a paradigm shift integrating clinical practice transformation and structural policy reforms. Neurological services should implement universal health literacy precautions as standard care: teach-back methods, pictorial medication schedules, routine literacy screening, and embedded patient navigators. Systems-level interventions must include rural telemedicine expansion, digital literacy programs for older adults, insurance simplification, and pharmaceutical subsidies. Addressing barriers as aggressively as neurological pathology itself requires a coordinated multi-sectoral response targeting social determinants through both healthcare delivery innovations and broader policy interventions.

## Data Availability

The raw data supporting the conclusions of this article will be made available by the authors, without undue reservation.
